# Hypolipidemic Activity and Antiatherosclerotic Effect of Polysaccharide of *Polygonatum sibiricum* in Rabbit Model and Related Cellular Mechanisms

**DOI:** 10.1155/2015/391065

**Published:** 2015-05-18

**Authors:** Jun-xuan Yang, Shen Wu, Xi-liang Huang, Xiao-quan Hu, Yi Zhang

**Affiliations:** ^1^School of Traditional Chinese Medicine, Chongqing Medical University, Chongqing 400016, China; ^2^Department of Laboratory Medicine, Guiyang Medical College, Guiyang, Guizhou 550004, China; ^3^Department of Cardiology, Fuzhou City First People's Hospital, Fuzhou, Jiangxi 344000, China; ^4^Department of Urology, Ningbo Zhenhai People's Hospital, Ningbo, Zhejiang 315202, China; ^5^Chongqing Institute for Food and Drug Control, Chongqing Engineering Center for Pharmaceutical Process and Quality Control, China

## Abstract

*Objective*. To evaluate the hypolipidemic activity and antiatherosclerotic effect of polysaccharide of *Polygonatum sibiricum* (PPGS), which is a kind of Chinese herbal medicine using the rhizome part of the whole herb. *Materials and Methods*. Thirty rabbits were divided into normal control group, model control group, and PPGS subgroups of 0.8, 1.6, and 3.2 mL/kg/day under random selection. In atherosclerosis model, the effects of PPGS on diverse blood lipids, foam cells number, and aortic morphology were evaluated. In the primary culture of endothelial cells (ECs), the activities of PPGS on both ECs proliferation and ECs injury were studied as well. *Results*. In atherosclerosis model, the hypolipidemic activities of PPGS were mainly focused on TC, LDL-C, and Lp(a). All changes on these factors were statistically significant compared with model group (*P* < 0.01), except TG and HDL-C. The intimal foam cell number of PPGS subgroups (0.8, 1.6, and 3.2 mL/kg/day) was significantly reduced than model control (*P* < 0.01). In the primary culture of endothelial cells (ECs), PPGS showed no effect on cell proliferation but preferred to protect EC from injury and apoptosis induced by H_2_O_2_ and lipopolysaccharide (LPS). *Discussion and Conclusion*. The antiatherosclerotic effect of PPGS may be supported by its hypolipidemic activities, improving aortic morphology, and reducing foam cells number and ECs injury.

## 1. Introduction

Plenty of studies showed that polysaccharide of* Polygonatum sibiricum* (PPGS), a popular Chinese herbal medicine in China, performed variety of medical effects such as anti-inflammation [1], antioxidation, and antiaging [2]. In Chinese database, there were only a few studies reporting the antiatherosclerotic effect of single dose of PPGS with no cellular mechanisms in detail [3, 4]. Therefore, the aims of this paper were to evaluate the possible cellular mechanism of PPGS on antiatherosclerosis based on endothelial cells (ECs) and smooth muscle cells (SMCs) and finally to further clarify the possible role of PPGS in the development of atherosclerosis process.

## 2. Materials and Methods

### 2.1. Animals

Thirty healthy male New Zealand rabbits in general grade with body mass of 1.9–2.4 kg were from Experimental Animal Center of Chongqing in China. Rabbits were kept in the cages with the condition of natural light, room temperature, relative humidity of (50 ± 3)%, and automatic ventilation. They were fed fixed dose of food with free access to drinking water. After the adaptive feeding for 7 d, animals were randomly divided into five groups (normal control group and model subgroups: model control group and PPGS subgroups (0.8, 1.6, and 3.2 mL/kg/day)). This study received the approval of Local Animal Ethics Advisory Committee.

### 2.2. Drugs, Chemicals, and Instruments

The PPGS extract (content of polysaccharide ≥90%) was purchased from Department of Preparation of Chongqing Chinese Medicine Hospital (Chongqing, China). The other reagents also included M199 medium and fetal bovine serum (Hyclone Co., Ltd., Utah, USA), II collagenase and trypsin (Invitrogen Corporation, Grand Island, USA), CCK-8 kit (Dojindo Laboratories, Kyushu, Japan), crystal violet (Sigma Chemical Co., St. Louis, USA), saline (for infusion, Kelun Co., Ltd., Sichuan, China), and neonatal umbilical cord (The Affiliated Hospital of Harbin Medical University, Harbin, China). The rabbit basal diet and relative high cholesterol diet were prepared from Experimental Animal Center of Chongqing Medical University (Chongqing, China) according to the reference study [5].

### 2.3. Hypolipidemic Activity and Antiatherosclerotic Effect in High Fat Diet-Induced Rabbit Model

Normal control group was fed with basal diet, while model subgroups were fed with high cholesterol diet. Each rabbit was given quantitative diet of 120 g/d, in which all were basal diet for normal control group and it was composed of 40 g/d high cholesterol diet and 80 g/d basic diet in model subgroups. The PPGS subgroups were also fed with different concentrations of PPGS (0.8, 1.6, and 3.2 mL/kg/day). The dose for animal model was converted from clinical dosage. During the feeding, high cholesterol diet was given firstly, and basal diet was supplemented with free access to water for 8 weeks.

After expiration of 8-week feeding with 10 h fasting, the venous blood was obtained for lipid levels testing (total cholesterol (TC), total triglycerides (TG), high-density lipoprotein (HDL-C), low-density lipoprotein (LDL-C), and lipoprotein (a) (Lp(a))). Then rabbits were sacrificed for study. In the experiments of HE staining, after conventional dehydration, paraffin sections were prepared for HE staining. Associated pathological changes of intima and adventitia under optical microscopy were recorded. Under certain magnification (20 × 10), eyepiece micrometer and hand control counters were applied to count foam cells number on 5 small lattices, taking the mean value of all slices to get cell number on each 1 mm^2^, which was seen as foam cell number per unit area of intima.

### 2.4. The Effects of PPGS on ECs and SMCs

Human umbilical vein endothelial cells (HUVECs) from umbilical cord were isolated by enzymatic digestion according to the method mentioned before [6]. Human umbilical artery smooth muscle cells (HUASMCs) were obtained by tissue adherent method [7, 8]. The digestion solution of 0.25% trypsin was prepared and added to serum medium. The supernatant was discarded after centrifugation; culture fluid was added to mix the cells and finally put them into culture flasks for cells growing at 37°C with 5% CO_2_. The third generations were used for the following experiments.

200 *μ*L of ECs or SMCs was seeded in 96-well plates according to the condition of 5 × 10^3^ cells/hole. After 24 h incubation, adherent cells were randomly divided into four groups: (1) control group, the culture medium (containing 20% fetal bovine serum), and (2) subgroups with different concentrations intervention of PPGS (25, 50, and 100 *μ*g/mL) in cultured cells. EC was cultured in drug-containing medium for 12, 24, and 48 h, while HUASMC was for 12, 18, and 24 h. Each dose subgroup was given five subholes. 10 *μ*L CCK-8 reagent was added into each hole for 2 h before the termination of culture. The absorbance of cell supernatant was detected at the wavelength of 450 nm, and cell culture medium was used as blank control of zero absorbance. In addition, the migration ability of HUASMC was determined by transwell migration chamber (8 *μ*m pore size) following the previous study [9]. The migration abilities of HUASMC in each group were estimated after the cells were fixed by 4% paraformaldehyde for 10 min, stained by crystal violet, and randomly selected under a fluorescence microscope with the magnification of 200x to count the number of cells migrating to the bottom of porous membrane.

### 2.5. The Effect on H_2_O_2_-Induced EC Injury

100 *μ*mol/L of H_2_O_2_ was selected as testing concentration. The main process was introduced in another study [10]. Each treatment group was given drug into medium with the final concentration of 10%. The normal control group was only administrated with medium. The model control group contained medium plus 100 *μ*mol/L of H_2_O_2_. The low dose treatment group was 0.3 mg/kg PPGS in cell medium plus 100 *μ*mol/L H_2_O_2_. The concentrations of PPGS in middle and high dose treatment group were 0.6 and 1.2 mg/kg, respectively. The cytokines (malondialdehyde (MDA) and superoxide dismutase (SOD)) were tested by related testing kits.

### 2.6. The Effect on Lipopolysaccharide- (LPS-) Induced EC Injury

After 24 h incubation, ECs were fused in adherent monolayer way [11]. The adherent cells were randomly divided into five groups the same as mentioned above. The suspension was abandoned and renewed; related drug was added into each group at the same time. After cell culture for 24 h at 37°C with 5% CO_2_, the old medium was abandoned. And 3 mL serum medium containing 5 *μ*g/mL of LPS was added into each group. The cells in each group were washed with PBS for 3 times 24 h later. Then 4% formaldehyde was used to fix them for 30 min. After washing for 3 times again with PBS solution, Hoechst 33258 fluorescent staining with the final concentration of 0.5 *μ*g/mL was done for 10 min in the dark at room temperature. The residual staining solution was discarded. The cellular washing was repeated again for 3 times. The fluorescence in the dark was observed in microscope at the wavelength of 350 nm. The cells in five different fields of picture view and cell apoptosis were calculated for each hole.

### 2.7. Statistical Analysis

Results were expressed in the form of mean ± SEM. Data were analyzed by one-way ANOVA, followed by Student's two-tailed *t*-test for comparison between two groups. *P* < 0.05 means statistically significant.

## 3. Result

### 3.1. Hypolipidemic Activity

After the treatment, the serum level of HDL-C and TG did not change basically. From Table 1, the results of other parameters were showed. All concentrations of PPGS were markedly effective on blood lipids control (*P* < 0.01).

### 3.2. The Results of HE Staining in Atherosclerotic Model

**Figure 1:**
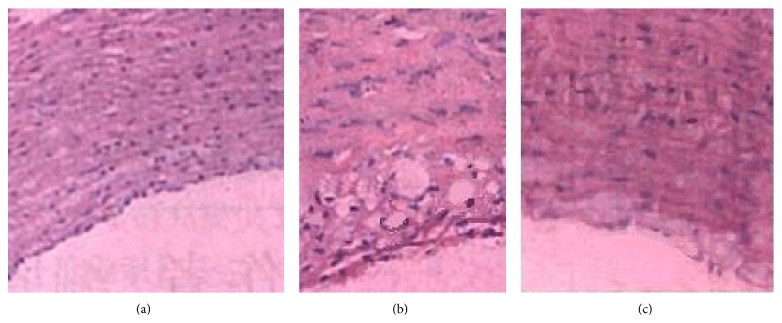
Microscope camera drawing of HE staining on rabbit thoracic aorta (200x): (a) normal group; (b) model control group; (c) 3.2 mL/kg/day PPGS group.

Aortic elastic membrane in normal diet group was integral. Endothelium was close to the internal elastic membrane arranged in neat rows, with smooth muscle and middle elastic membrane arranged in parallel. In model control group, aortic intima was significantly thickening with a large accumulation of foam cells. ECs were falling off or loosely attached to the membrane surface. Intimal lesions had extensively pathological changes with collagen fiber glass. Elastic fiber was ruptured and disappeared as well. In PPGS subgroups, compared with the thickening degree of aortic intima in model control group, subendothelial gap was increased with visible foam cells aggregation, but foam cells number was significantly less than model control group. The structure of medial membrane was basically integral with SMCs in the same pole, as shown in Figure 1 (200x).

### 3.3. The Effects of PPGS on Foam Cell Number in Atherosclerotic Model

**Figure 2:**
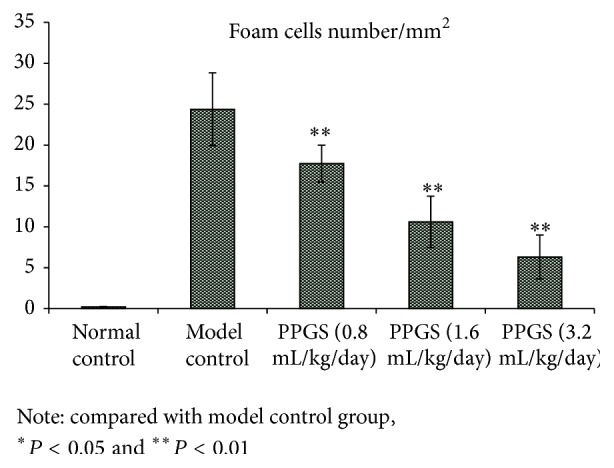
The effects of PPGS after being administered continuously for 8 weeks on foam cells number in atherosclerosis rabbit model (*n* = 6).

As shown in Figure 2, PPGS could significantly reduce the foam cells number in atherosclerosis model.

### 3.4. The Effect of PPGS on EC Proliferation

ECs were cultured in drug-containing medium for 12, 24, and 48 h. After that, the OD values of culture supernatants in each group were recorded. The results demonstrated that, compared with control group, there was no obvious effect on ECs proliferation for PPGS subgroups (no data shown).

### 3.5. The Effect of PPGS on H_2_O_2_-Induced EC Injury

**Figure 3:**
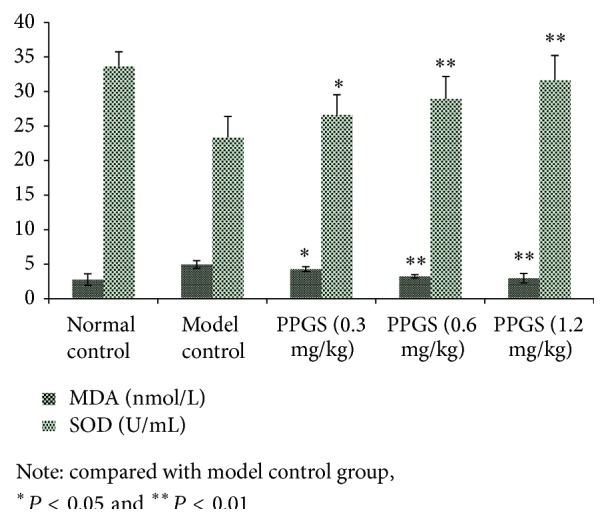
The impact of PPGS on EC injury and related cytokines releasing (*n* = 6).

The concentration of cytokines (MDA and SOD) expressed by EC can indirectly respond to the injury degree of EC induced by H_2_O_2_. Therefore, Figure 3 showed some data in detail to prove the protection effect of PPGS on EC injury. MDA rising meant that EC was damaged under the condition of H_2_O_2_ oxidation. Meanwhile, the activity of SOD was also decreased. PPGS had the protection effect to reverse oxidative injury.

### 3.6. The Effect on LPS-Induced EC Injury

**Figure 4:**
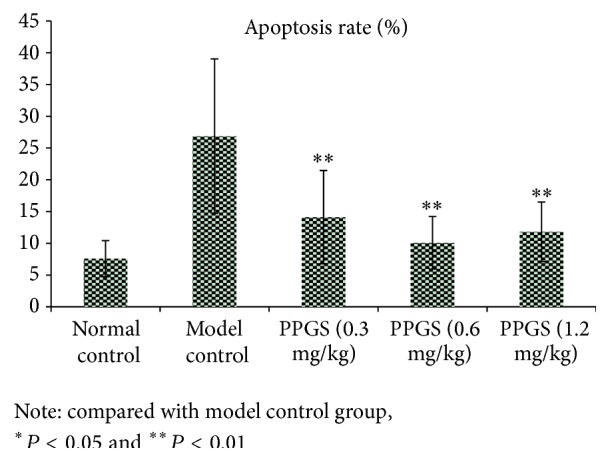
The impact of PPGS on EC injury and apoptotic condition (*n* = 6).

After Hoechst 33258 fluorescent staining, the cell apoptosis could be identified with different color.  Figure 4 identified that PPGS had protection effect on LPS-induced cell apoptosis.

### 3.7. The Effect of PPGS on SMCs Proliferation

**Figure 5:**
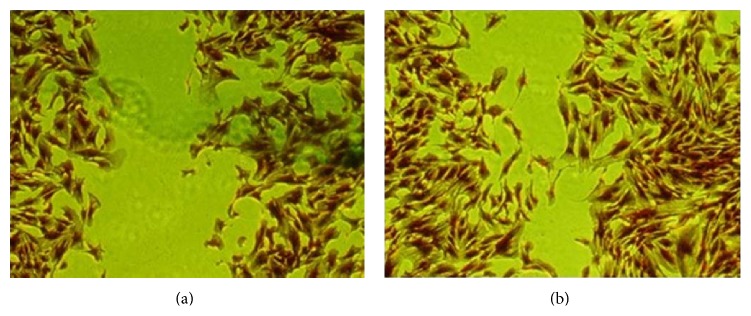
The crystal violet staining of HUASMCs on each group: (a) 1.2 mg/kg PPGS subgroup and (b) control group (200x).

SMCs were cultured in drug-containing medium for 12, 18, and 24 h. The OD values of culture supernatants in each group were tested (Table 2). The results demonstrated that, compared with control group, there was obvious inhibition effect on SMCs proliferation for PPGS subgroups (0.6 and 1.2 mg/kg), which also showed significant prevention activity on SMCs migration (*P* < 0.05) (Table 2 and Figure 5).

## 4. Discussion

Inflammatory mediators may play an important role in the occurrence and development process of atherosclerosis. This study evaluated the effect of PPGS on high cholesterol-bearing atherosclerosis model. The results showed that, after the administration of PPGS, the blood levels of TC, LDL-C, and Lp(a) were decreased but not TG or HDL-C. It might mean that the hypolipidemic activities of PPGS mainly focused on hypercholesterolemia. The LDL-C was also a kind of lipoprotein composed of protein and cholesterol and was sensitive to the concentration of cholesterol in blood.

For the ECs, there was no evidence showing that PPGS had an effect directly on the ECs proliferation. But it had some positive interaction with the ECs apoptosis and injury. The H_2_O_2_-induced cell injury may result into necrosis based on strong oxidation reaction. And LPS was more focused on cell apoptosis by inhibiting NOS activity and NO content both inside and outside of cell, but increasing the intracellular ROS level [12]. For another distinction based on physiological response, H_2_O_2_-induced cell injury may lead to inflammation and thrombosis for its acute oxidation. But the chronic oxidation effect of LPS may finally lead to atherosclerosis and plaque instability [13]. So the protection effects of PPGS on both apoptosis and necrosis of ECs were really impressive, which also indicated the primary mechanism of PPGS on atherosclerosis for the first time.

It was learnt that PPGS was composed of single monosaccharide fructose with the relative molecular mass (Mr) of 7247 [14]. In this study, the purity of PPGS was up to 90% and was good enough to evaluate the hypolipidemic activity and antiatherosclerotic effect of PPGS initially. However, these activities in long-term period were still unknown, which might be more beneficial for clinical recommendation. Besides, further studies on more animal models with different injury degree of blood vessels were still needed.

Therefore, PPGS might play important roles on antiatherosclerosis in two different levels: (1) one is suppressing the level of blood lipids directly, (2) and another is to protect ECs from apoptosis and necrosis indirectly in cellular level. Thereafter, its activities on smooth muscle cells or other cytokines were also warranted. In one word, they all resulted into the antiatherosclerosis effect of PPGS in cellular level and animal model.

## Figures and Tables

**Table 1:** The hypolipidemic effects of PPGS in atherosclerosis rabbit model (*n* = 6).

Groups	TC		LDL-C		Lp(a)	
	Before	After	Before	After	Before	After
Normal control	1.26 ± 0.25	1.40 ± 0.33	1.18 ± 0.26	1.26 ± 0.17	50.41 ± 10.33	67.32 ± 15.13
Model control	1.24 ± 0.31	12.18 ± 2.40	1.17 ± 0.24	10.46 ± 1.53	47.35 ± 15.16	643.72 ± 151.69
PPGS (0.8 mL/kg/day)	1.31 ± 0.40	7.82 ± 4.13^∗∗^	1.20 ± 0.31	6.42 ± 3.48^∗∗^	49.52 ± 8.82	81.40 ± 26.73^∗∗^
PPGS (1.6 mL/kg/day)	1.38 ± 0.41	5.81 ± 1.92^∗∗^	1.25 ± 0.27	4.61 ± 1.56^∗∗^	51.9 ± 14.83	47.36 ± 15.39^∗∗^
PPGS (3.2 mL/kg/day)	1.32 ± 0.37	4.5 ± 2.11^∗∗^	1.22 ± 0.25	3.45 ± 0.73^∗∗^	46.68 ± 14.81	33.52 ± 12.68^∗∗^

Note: compared with model control group, ^∗∗^ *P* < 0.01.

**Table 2:** The effects of PPGS on HUASMCs proliferation and migration (*n* = 6).

Groups	OD values			Cells migration number 24 h later (*n* = 3)
	12 h	24 h	48 h	
Normal control	0.186 ± 0.024	0.202 ± 0.022	0.215 ± 0.027	39.1 ± 4.2
PPGS (0.3 mg/kg)	0.172 ± 0.020	0.171 ± 0.024^∗^	0.177 ± 0.019^∗^	35.8 ± 4.6
PPGS (0.6 mg/kg)	0.159 ± 0.022^∗^	0.162 ± 0.017^∗∗^	0.169 ± 0.015^∗∗^	33.2 ± 4.3^∗^
PPGS (1.2 mg/kg)	0.138 ± 0.015^∗^	0.141 ± 0.015^∗∗^	0.155 ± 0.018^∗∗^	30.6 ± 4.1^∗∗^

Note: compared with model control group, ^∗^ *P* < 0.05 and ^∗∗^ *P* < 0.01.
